# Endoscopic esophageal cicatricotomy assisted by submucosal injection for the treatment of esophageal stenosis after endoscopic submucosal dissection

**DOI:** 10.1055/a-2210-0428

**Published:** 2023-12-13

**Authors:** Wen Ji, Senrong Luo, Shijun Wang, Huan He, Weiqing Chen

**Affiliations:** 1 605425 Department of Gastroenterology, Chongqing University Cancer Hospital, Chongqing, China

Esophageal stenosis is a common complication after endoscopic submucosal dissection (ESD) for early esophageal cancer 1 2 . Post-ESD esophageal stenosis is considered to be related to scar tissue proliferation and fibrosis during the healing process 3 . Symptom relief can be achieved through dilation and radial incision in 83% 4 . However, these invasive strategies do not allow visual control of the operation, and it is difficult to control complications such as bleeding or perforation caused by these treatments.

A 77-year-old patient with esophageal stricture after ESD for early esophageal cancer underwent three endoscopic esophageal stricture expansion procedures over 6 months. Endoscopic examination showed that the patient had irregular stenosis in the middle esophagus, which the gastroscope could not pass. Under white-light and narrow-band imaging ( Fig. 1 ), the approximate starting position of the scar could be determined. Submucosal injection and submucosal incision were then performed on the oral side of the scar ( Fig. 2 , Video 1 ). Under the blue background of the submucosal injectate, which contained indigo carmine, the white scar was clearly distinguishable. By using this contrast in visual appearance, it was easy to accurately identify and release all scar tissue using submucosal incision under direct visualization, until the gastroscope could pass smoothly through the esophageal stricture ( Fig. 3 ). The incision length was 27–31 cm from the incisors, and the operation time was about 30 minutes. There were no bleeding or perforation complications during the process.

**Fig. 1:**
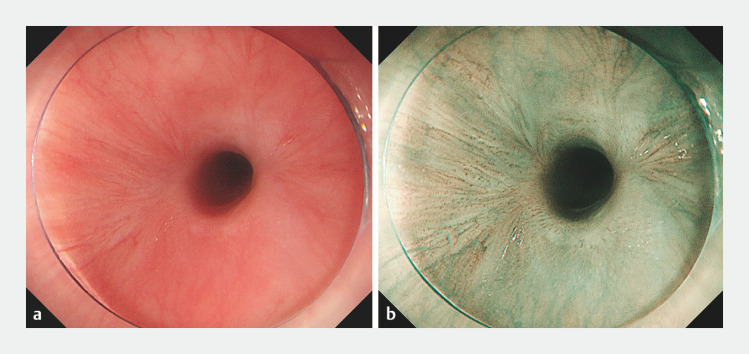
Imaging of the stricture. **a** White-light imaging. **b** Narrow-band imaging.

**Fig. 2:**
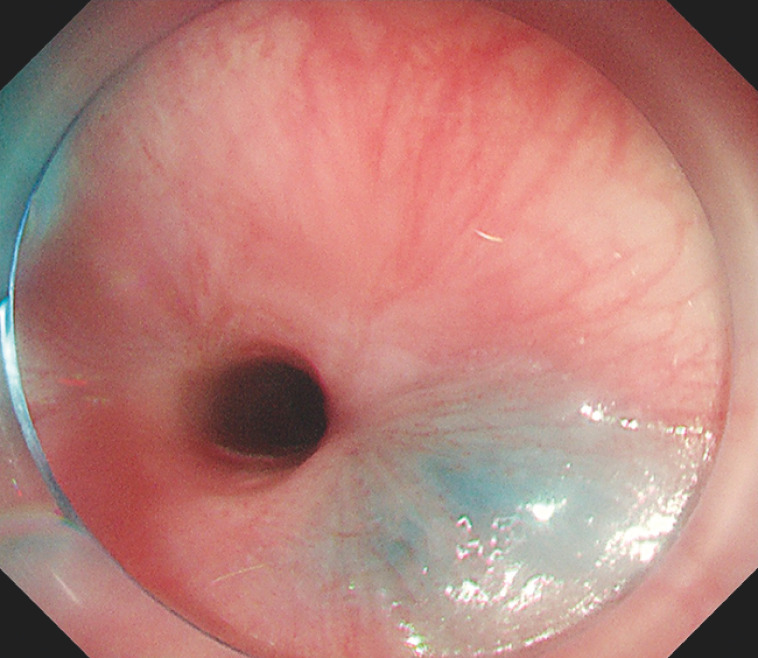
Submucosal injection and incision were performed on the oral side of the stricture.

Endoscopic cicatricotomy of the esophagus, assisted by submucosal injection.Video 1

**Fig. 3:**
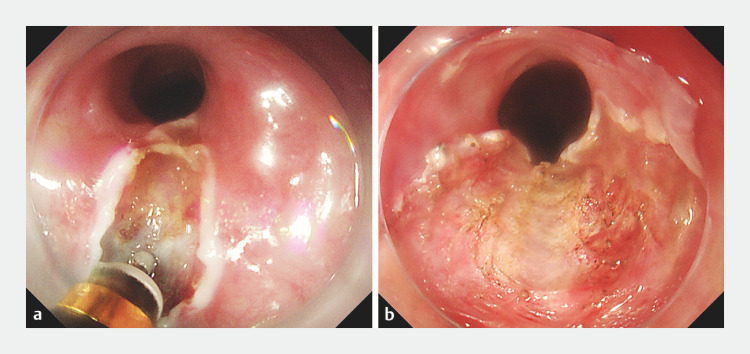
Treatment of the esophageal stricture. **a** Submucosal incision and release of all scar tissue under direct visualization. **b** After treatment, the gastroscope could pass through the esophagus.

The esophagus that has undergone multiple expansions will have multiple complex fibrotic areas and severe submucosal adhesions. Submucosal injection-assisted cicatricotomy can aid visualization of the scar and submucosa during the operation, allowing the endoscopists to accurately control the incision site and depth, minimize the damage to normal mucosa, and successfully reduce the risk of perforation and bleeding.

Endoscopy_UCTN_Code_TTT_1AO_2AH

## Notes

Endoscopy E-Videos https://eref.thieme.de/e-videos
*E-Videos* is an open access online section of the journal *Endoscopy* , reporting on interesting cases and new techniques in gastroenterological endoscopy. All papers include a high-quality video and are published with a Creative Commons CC-BY license. Endoscopy E-Videos qualify for HINARI discounts and waivers and eligibility is automatically checked during the submission process. We grant 100% waivers to articles whose corresponding authors are based in Group A countries and 50% waivers to those who are based in Group B countries as classified by Research4Life (see: https://www.research4life.org/access/eligibility/ ). This section has its own submission website at https://mc.manuscriptcentral.com/e-videos .
